# Altering the Architecture of Tissue Engineered Hypertrophic Cartilaginous Grafts Facilitates Vascularisation and Accelerates Mineralisation

**DOI:** 10.1371/journal.pone.0090716

**Published:** 2014-03-04

**Authors:** Eamon J. Sheehy, Tatiana Vinardell, Mary E. Toner, Conor T. Buckley, Daniel J. Kelly

**Affiliations:** 1 Trinity Centre for Bioengineering, Trinity Biomedical Sciences Institute, Trinity College Dublin, Dublin, Ireland; 2 Department of Mechanical and Manufacturing Engineering, School of Engineering, Trinity College Dublin, Dublin, Ireland; 3 Advanced Materials and Bioengineering Research Centre (AMBER), Trinity College Dublin, Dublin, Ireland; 4 School of Agriculture and Food Science, University College Dublin, Belfield, Dublin, Ireland; 5 Department of Pathology, School of Dental Science, Trinity College Dublin, Dublin, Ireland; University of California at Davis, United States of America

## Abstract

Cartilaginous tissues engineered using mesenchymal stem cells (MSCs) can be leveraged to generate bone *in vivo* by executing an endochondral program, leading to increased interest in the use of such hypertrophic grafts for the regeneration of osseous defects. During normal skeletogenesis, canals within the developing hypertrophic cartilage play a key role in facilitating endochondral ossification. Inspired by this developmental feature, the objective of this study was to promote endochondral ossification of an engineered cartilaginous construct through modification of scaffold architecture. Our hypothesis was that the introduction of channels into MSC-seeded hydrogels would firstly facilitate the *in vitro* development of scaled-up hypertrophic cartilaginous tissues, and secondly would accelerate vascularisation and mineralisation of the graft *in vivo*. MSCs were encapsulated into hydrogels containing either an array of micro-channels, or into non-channelled ‘solid’ controls, and maintained in culture conditions known to promote a hypertrophic cartilaginous phenotype. Solid constructs accumulated significantly more sGAG and collagen *in vitro*, while channelled constructs accumulated significantly more calcium. *In vivo*, the channels acted as conduits for vascularisation and accelerated mineralisation of the engineered graft. Cartilaginous tissue within the channels underwent endochondral ossification, producing lamellar bone surrounding a hematopoietic marrow component. This study highlights the potential of utilising engineering methodologies, inspired by developmental skeletal processes, in order to enhance endochondral bone regeneration strategies.

## Introduction

The goal of tissue engineering is to replace or regenerate damaged tissues through the combination of cells, three-dimensional scaffolds, and signalling molecules [1], [2]. Bone tissue engineering has, thus far, generally focussed on the direct osteoblastic differentiation of mesenchymal stem cells (MSCs), in a process resembling intramembranous ossification [3]. However, the success of this approach to bone regeneration has been hampered by insufficient blood vessel infiltration, preventing the necessary delivery of oxygen and nutrients to the engineered graft [4], [5]. Recently an endochondral approach to bone tissue engineering, which involves remodelling of an intermediary hypertrophic cartilaginous template [6]–[14], has been proposed as an alternative to direct intramembranous ossification for bone regeneration using MSCs. Chondrogenically primed bone marrow derived MSCs have an inherent tendency to undergo hypertrophy [15], an undesirable attribute in MSC-based articular cartilage repair therapies, but one which may be harnessed for use in endochondral bone tissue engineering strategies. Hypertrophic chondrocytes are equipped to survive the hypoxic environment a tissue engineered graft will experience once implanted *in vivo* [14]. Furthermore, cells undergoing hypertrophy release pro-angiogenic factors, such as vascular endothelial growth factor, to facilitate the conversion of avascular tissue to vascularised tissue [16]. The endochondral approach to bone regeneration may therefore circumvent many of the issues associated with the traditional intramembranous method; however, in order to be used to repair large bone defects, this approach first requires strategies to engineer scaled-up hypertrophic cartilage. This may be challenging, as recent attempts to generate large hypertrophic constructs using MSC seeded collagen scaffolds have demonstrated the formation of a core region devoid of cells and matrix [12].

Such ‘scaling up’ of engineered grafts, and the associated issue of core degradation, is a well-documented challenge in the field of tissue engineering [17]. Strategies to promote nutrient supply and waste removal include the use of bioreactors [18]–[21], modification of scaffold architectures [22], [23], or a combination of both [24]–[26]. An alternative approach might be to recapitulate the mechanisms adopted during skeletogenesis to provide nutrients to the cartilaginous templates. During normal bone development, canals within the developing hypertrophic cartilage have been identified [27], [28]. These canals play an important role in supplying nutrients to the developing cartilage [29]. Furthermore, endochondral ossification is dependent on neo-vascularisation of the cartilaginous template, with the in-growth of blood vessels via these cartilage canals identified as a key event during bone development [29]. Finally, these canals act as conduits for the migration of osteogenic cells into the cartilaginous template which in turn lay down new bone matrix [30]. Executing a developmental engineering paradigm [31], directed at mimicking the structure and function of the cartilage canal network, may therefore be a powerful tool in endochondral bone tissue engineering strategies.

Inspired by the cartilage canal network observed during endochondral skeletogenesis, we hypothesised that the introduction of channels into hypertrophic cartilaginous constructs would firstly facilitate extracellular matrix synthesis and the *in vitro* development of the construct, and secondly would accelerate mineralisation and vascularisation of the graft once implanted *in vivo*. To assess the influence of scaffold architecture on *in vitro* graft development, bone marrow derived MSCs were encapsulated in agarose hydrogels either containing an array of micro-channels (termed ‘channelled’), or in non-channelled (termed ‘solid’) controls, and cultured long-term (up to 10 weeks) to undergo hypertrophic chondrogenic differentiation. Furthermore, to test the efficacy of channelled architectures to enhance mineralisation and vascularisation *in vivo,* channelled and solid constructs were also subjected to a shorter period of hypertrophic priming (6 weeks) prior to subcutaneous implantation in nude mice. Constructs were harvested at 4 and 8 weeks post-implantation to test our hypothesis that graft architecture would influence mineralisation and vascularisation of the engineered hypertrophic tissue.

## Methods

### Experimental Design

The first phase of this study investigated the *in vitro* development of engineered cartilaginous constructs undergoing long- term hypertrophic chondrogenesis. MSCs were encapsulated in solid and channelled hydrogels, cultured in chondrogenic conditions (further details below), for a period of 5 weeks. Thereafter constructs were switched to hypertrophic conditions for an additional 5 weeks, resulting in a total *in vitro* culture period of 10 weeks. The second phase of this study investigated the potential of engineered hypertrophic cartilaginous constructs to undergo mineralisation and vascularisation *in vivo*. MSCs were encapsulated in solid and channelled constructs and cultured in chondrogenic conditions for a period of 5 weeks, as per phase 1. Thereafter constructs received an additional week in hypertrophic conditions (6 weeks total *in vitro* priming) prior to subcutaneous implantation in nude mice. Constructs were harvested at 4 and 8 weeks post-implantation.

### Cell Isolation and Expansion

Porcine bone marrow derived MSCs (4 months old) from the femoral shaft were isolated aseptically and expanded according to a modified method for human MSCs [32] in high glucose Dulbecco’s modified eagle’s medium (DMEM) GlutaMAX supplemented with 10% v/v foetal bovine serum and 100 U/mL penicillin –100 µg/mL streptomycin (all Gibco, Biosciences, Dublin Ireland) at 20% pO_2_. Following colony formation, MSCs were trypsinised, counted, seeded at density of 5×10^3^ cells/cm^2^, and expanded to passage 1 (P1). MSCs underwent 15 population doublings prior to encapsulation within hydrogels.

### Cell Encapsulation in Solid and Channelled Agarose Hydrogels

At the end of P1 MSCs were suspended in 2% agarose at a density of 20×10^6^ cells/mL. The agarose cell suspension was cast in a stainless steel mould to produce regular solid (non-channelled) cylindrical constructs (Ø5 mm×3 mm). Channelled cylindrical constructs were fabricated utilising a pillared polydimethylsiloxane array structure [24] inserted into a cylindrical Tufset mould to produce a unidirectional 4×3 channelled array in the longitudinal direction with diameters of 500 µm and a centre-centre spacing of 1 mm.

### Chondrogenic and Hypertrophic Culture Conditions

The chondrogenic conditions applied in this study are defined as culture at 5% pO_2_ in a chondrogenic medium consisting of high glucose DMEM GlutaMAX supplemented with 100 U/mL penicillin/streptomycin (both Gibco), 100 µg/mL sodium pyruvate, 40 µg/mL L-proline, 50 µg/mL L-ascorbic acid-2-phosphate, 4.7 µg/mL linoleic acid, 1.5 mg/mL bovine serum albumine, 1×insulin–transferrin–selenium, 100 nM dexamethasone (all from Sigma-Aldrich) and 10 ng/mL of human transforming growth factor-β3 (TGF-β3) (Prospec-Tany TechnoGene Ltd., Israel) [33]. The hypertrophic conditions applied are defined as culture at 20% pO_2_ in a hypertrophic medium consisting of high glucose DMEM GlutaMAX supplemented with 100 U/mL penicillin/streptomycin, 100 µg/mL sodium pyruvate, 40 µg/mL L-proline, 50 µg/mL L-ascorbic acid-2-phosphate, 4.7 µg/mL linoleic acid, 1.5 mg/mL bovine serum albumine, 1×insulin–transferrin–selenium, 1 nM dexamethasone, 1 nM L-thyroxine and 20 µg/mL β-glycerophosphate (both Sigma-Aldrich) [13], [34].

### In vivo Subcutaneous Transplantation

In phase 2 of the study, following 6 weeks *in vitro* priming, MSC- seeded solid and channelled constructs were implanted subcutaneously into the back of nude mice (Balb/c; Harlan, UK) as previously described [34]. Briefly, two subcutaneous pockets were made along the central line of the spine, one at the shoulders and the other at the hips, and into each pocket three constructs were inserted. Nine constructs were implanted per each experimental group. Mice were sacrificed at 4 and 8 weeks post-implantation by CO_2_ inhalation. In addition, three acelluar solid and three acellular channelled hydrogels were implanted as controls and harvested at 8 weeks post-implantation. These controls were not primed in chondrogenic and hypertrophic culture conditions before transplantation. A total of 7 mice were used for the experiment. The animal protocol was reviewed and approved by the ethics committee of Trinity College Dublin.

### Biochemical Analysis

The biochemical content of constructs was analysed at each time point. Prior to biochemical analysis, constructs were sliced in half, washed in phosphate buffered saline (PBS), weighed, and frozen for subsequent assessment. Half of each construct was digested with papain (125 µg/mL) in 0.1 M sodium acetate, 5 mM L-cysteine-HCL, 0.05 M ethylenediaminetetraacetic acid (EDTA), pH 6.0 (all from Sigma-Aldrich) at 60°C and 10 rpm for 18 h. DNA content was quantified using the Hoechst Bisbenzimide 33258 dye assay, with a calf thymus DNA standard. Proteoglycan content was estimated by quantifying the amount of sulphated glycosaminoglycan (sGAG) using the dimethylmethylene blue dye-binding assay (Blyscan, Biocolor Ltd., Northern Ireland), with a chondroitin sulphate standard. Total collagen content was determined by measuring the hydroxyproline content, using a hydroxyproline-to-collagen ratio of 1∶7.69 [35], [36]. The other half was digested in 1 M hydrochloric acid (HCL) (Sigma-Aldrich) at 60°C and 10 rpm for 18 h. The calcium content was determined using a Sentinel Calcium kit (Alpha Laboratories Ltd, Uk). 3–4 constructs were analysed biochemically per each *in vitro* experimental group. 4–6 constructs were analysed biochemically per each *in vivo* experimental group.

### Histology and Immunohistochemistry

At each time point samples were fixed in 4% paraformaldehyde overnight, dehydrated in a graded series of ethanols, embedded in paraffin wax, sectioned at 8 µm and affixed to microscope slides. Samples harvested at 8 weeks post- *in vivo* implantation were decalcified in EDTA for 3–4 days prior to wax embedding. The sections were stained with haematoxylin and eosin (H&E), 1% alcian blue 8GX in 0.1 M HCL to assess sGAG content with a counter stain of nuclear fast red to assess cellular distribution, picro-sirius red to assess collagen distribution, and 1% alizarin red to assess calcium accumulation (all Sigma-Aldrich). Collagen types I, II and X were evaluated using a standard immunohistochemical technique; briefly, sections were treated with peroxidase, followed by treatment with chondroitinase ABC (Sigma-Aldrich) in a humidified environment at 37°C to enhance permeability of the extracellular matrix. Sections were incubated with goat serum to block non-specific sites and collagen type I (ab6308, 1∶400; 1 mg/mL), collagen type II (ab3092, 1∶100; 1 mg/mL) or collagen type X (ab49945, 1∶200; 1.4 mg/mL) primary antibodies (mouse monoclonal, Abcam, Cambridge, UK) were applied for 1 h at room temperature. Next, the secondary antibody (Anti-Mouse IgG biotin conjugate, 1∶200; 2.1 mg/mL) (Sigma-Aldrich) was added for 1 h followed by incubation with ABC reagent (Vectastain PK-400, Vector Labs, Peterborough, UK) for 45 min. Finally sections were developed with DAB peroxidase (Vector Labs) for 5 min. Positive and negative controls were included in the immunohistochemistry staining protocol for each batch. Terminal deoxynucleotidyl Transferase (TdT) cells were identified on the fully automated IHC Leica BOND-MAX (Leica biosystems). Briefly, sections were incubated with a mouse monoclonal TdT Clone SEN28 ready to use primary antibody (PA0339) (Leica biosystems). Heat mediated antigen retrieval was performed with epiptope retrieval solution 2 (AR9640) for 10 min (Leica biosystems). Thereafter, visualisation of the antibody was performed with the Bond Polymer Refine Detection kit (DS9800) (Leica biosystems). Heat mediated antigen retrieval was performed on CD31 sections with sodium citrate solution (Sigma-Alrich) for 20 min, and incubated with goat serum, biotin, and avidin, to block non-specific sites, and the CD31 (ab28364, 1∶50; 0.2 mg/mL) primary antibody (Rabbit polyclonal, Abcam, Cambridge, UK) was applied overnight at 4°C. Thereafter sections were treated with peroxidase followed by the secondary antibody (ab97051, goat polyclonal secondary antibody to Rabbit IgG, 1∶200, 1 mg/mL, Abcam, Cambridge, UK) for 1 h, ABC reagent for 45 min, and developed with DAB peroxidase for 5 min. (both Vector Labs). Histological images are taken from two representative constructs.

### Micro-computed Tomography

Micro-computed tomography (µCT) scans were carried out on constructs using a Scanco Medical 40 µCT system (Scanco Medical, Bassersdorf, Switzerland) in order to quantify mineral content and assess mineral distribution. In phase 1 of the experiment, constructs were scanned at the end of the 10 week *in vitro* culture period. In phase 2, constructs were scanned at 4 and 8 weeks post-implantation. Constructs were scanned in PBS, at a voxel resolution of 12 µm, a voltage of 70 kVp, and a current of 114 µA. Circular contours were drawn in an anti-clockwise direction around the periphery of the constructs. Additionally, circular constructs were drawn in a clockwise direction around the perimeter of the channels, so as to exclude the area with the channels from the analysis. A Gaussian filter (sigma = 0.8, support = 1) was used to suppress noise and a global threshold corresponding to a density of 172.6 mg hydroxyapatite/cm^3^ was applied. This threshold was selected by visual inspection of individual scan slices so as to include mineralised tissue and exclude non-mineralised tissue. 3D evaluation was carried out on the segmented images to determine mineral volume and to reconstruct a 3D image. The variance of mineralisation with depth through the constructs was analysed qualitatively by examining sections ∼0.75 mm of depth from the top of the construct (quarter section), and ∼1.5 mm of depth from the top of the construct (centre section). 10 slices were compiled per section, corresponding to a thickness of 120 µm.

### Statistical Analysis

All statistical analyses were carried out using Minitab 15.1. Results are reported as mean ± standard deviation. The number of constructs analysed per group are provided in the figure legends. Groups were analysed by a general linear model for analysis of variance with groups of factors. Tukey’s test was used to compare conditions. Anderson-Darling normality tests were conducted on residuals to confirm a normal distribution. Non-normal data was transformed using the Box-Cox procedure. Any non-normal data which the Box-Cox procedure could not find a suitable transformation for was transformed using the Johnson procedure. Significance was accepted at a level of *p*<0.05.

## Results

### Chondrogenically Primed MSCs can be Stimulated in vitro to Produce a Calcified Cartilaginous Tissue within Solid and Channelled Hydrogels

**Figure 1:**
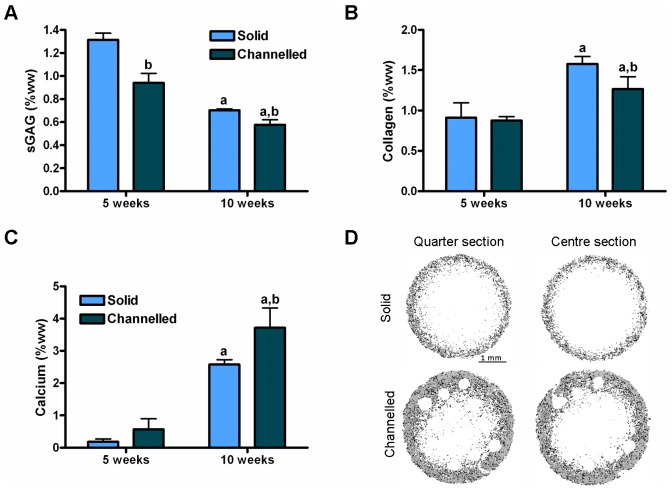
Biochemical and µCT analysis of constructs after 10 weeks of *in vitro* culture. Solid and channelled constructs were subjected to 5 weeks culture in chondrogenic conditions, followed by an additional 5 weeks culture in hypertrophic conditions. (A) sGAG, (B) collagen, (C) calcium (% wet weight) accumulation of solid and channelled constructs after 5 and 10 weeks of *in vitro* culture. 3–4 constructs per group were analysed. Significance *p*<0.05: a vs. 5 weeks, b vs. solid constructs. (D) µCT analysis of solid and channelled constructs after 10 weeks *in vitro* culture. Quarter section corresponds to a region ∼0.75 mm into the depth of the construct. Centre section corresponds to a region ∼1.5 mm into the depth of the construct. Sections correspond to a thickness of 120 µm. Scale bar is consistent across all images. Images are representative of 3 constructs analysed.

MSCs were encapsulated in solid and channelled hydrogels and were subjected to chondrogenic culture conditions for a period of 5 weeks, followed by an additional 5 weeks in hypertrophic culture conditions, resulting in a total *in vitro* culture period of 10 weeks. Solid MSC-seeded hydrogels accumulated significantly more sGAG compared to channelled MSC- seeded hydrogels after 5 weeks (1.31±0.06 vs. 0.94±0.08%ww, *p*<0.0001) and 10 weeks (0.70±0.01 vs. 0.58±0.04%ww, *p = *0.0304) of *in vitro* culture, see Fig. 1(A). Both solid and channelled constructs show a significant reduction in sGAG after 10 weeks compared to their levels at 5 weeks. Both collagen and calcium accumulation increased with time in culture for both solid and channelled constructs, with greater levels of collagen observed in solid constructs after 10 weeks of culture (1.58±0.09 vs. 1.27±0.15%ww, *p* = 0.03), see Fig. 1(B). Channelled constructs accumulated significantly more calcium than solid constructs after 10 weeks in culture (3.72±0.61 vs. 2.58±0.15%ww, *p* = 0.0035), see Fig. 1(C). µCT analysis confirmed the enhancement of mineralisation in channelled constructs, see Fig. 1(D). Both solid and channelled constructs mineralised preferentially around their periphery, with a reduction in mineralisation observed with depth through the constructs.

### Channelled Architectures Accelerate in vivo Mineralisation of MSC-seeded Hydrogels

**Figure 2:**
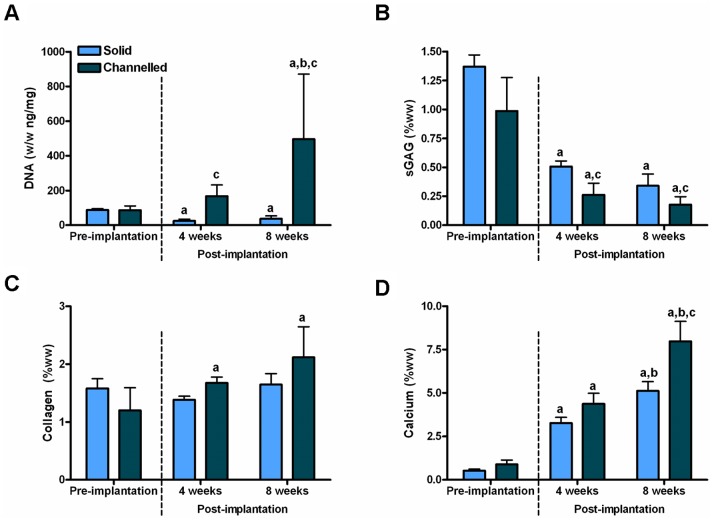
Biochemical analysis of constructs pre-implantation and post-implantation. Solid and channelled constructs were subjected to 5 weeks culture in chondrogenic conditions, followed by 1 week of culture in hypertrophic conditions, and then implanted subcutaneously into nude mice to be harvested at 4 weeks and 8 weeks post-implantation. (A) DNA content, normalised to mg wet weight. (B) sGAG, (C) collagen and (D) calcium content (% wet weight). Significance *p*<0.05: a vs pre-implantation, b vs. 4 weeks post-implantation, c vs. solid constructs. 3–4 constructs per group were analysed pre-implantation. 4–6 constructs per group were analysed post-implantation.

MSC- seeded solid and channelled hydrogels underwent 5 weeks of culture in chondrogenic conditions, followed by an additional week of culture in hypertrophic conditions, prior to subcutaneous implantation in nude mice. Constructs were harvested at 4 and 8 weeks post-implantation. The DNA content of solid constructs 4 and 8 weeks post-implantation were lower than pre- implantation levels, whereas the DNA content of channelled constructs was higher after 4 weeks *in vivo* compared to pre-implantation levels, and further increased after 8 weeks *in vivo*, see Fig. 2(A). The sGAG content of both solid and channelled constructs was lower 4 and 8 weeks post-implantation compared to pre-implantation levels, with solid constructs maintaining significantly higher levels of sGAG at both post-implantation time points compared to channelled constructs (0.34±0.1 vs. 0.18±0.07%ww at 8 weeks post-implantation, *p* = 0.0001), see Fig. 2(B). Prior to implantation, collagen accumulation was similar in solid and channelled constructs. Collagen levels did not change in solid constructs post-implantation, whereas collagen accumulation was significantly higher in channelled constructs at 4 and 8 weeks post implantation compared to pre-implantation levels (2.12±0.53 vs. 1.2±0.39%ww, 8 weeks post-implantation vs. pre-implantation, *p* = 0.0005), see Fig. 2(C). The calcium content of solid and channelled constructs increased at 4 weeks post-implantation compared to pre-implantation, with a further increase evident at 8 weeks post-implantation compared to 4 weeks post-implantation, see Fig. 2(D). Calcium accumulation in channelled constructs showed a trend towards a significant increase, as compared to solid constructs, at 4 weeks post-implantation (4.37±0.61 vs. 3.27±0.33%ww, *p* = 0.0593), with this difference becoming significant at 8 weeks post-implantation (7.97±1.15 vs. 5.12±0.53%ww, *p*<0.0001).

**Figure 3:**
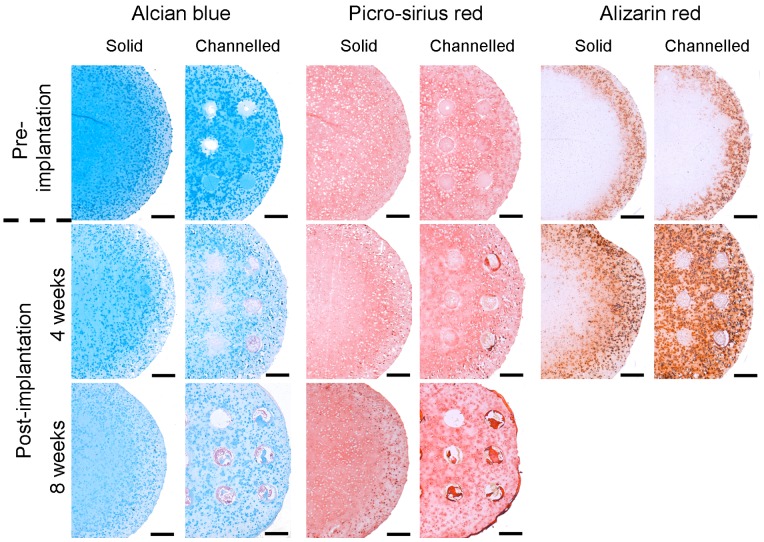
Histology of constructs pre-implantation and post-implantation. Solid and channelled constructs were subjected to 5 weeks culture in chondrogenic conditions, followed by 1 week of culture in hypertrophic conditions, and then implanted subcutaneously into nude mice to be harvested at 4 weeks and 8 weeks post-implantation. Constructs were stained with alcian blue, picro-sirius red and alizarin red to assess sGAG, collagen, and calcium accumulation respectively. 8 weeks post-implantation samples were decalcified prior to histological analysis, hence alizarin red staining was not undertaken at this time point. Images show half the construct. Scale bars are 500 µm.

Histological analysis of constructs, pre- and post- implantation, was carried out using alcian blue, picro-sirius red and alizarin red staining to assess the spatial distribution of sGAG, collagen, and calcium deposition respectively, see Fig. 3. Prior to implantation, solid and channelled constructs stained positively and homogenously for alcian blue and picro-sirius red, whereas both constructs only stained positively for alizarin red around their periphery. At 4 weeks post-implantation, solid and channelled constructs stained weakly for alcian blue with channels beginning to show positive staining for nuclear fast red. An increased number of channels stained positive for nuclear fast red at 8 weeks post-implantation. Tissue that had filled the channels also stained intensely for picro-sirius red at 8 weeks post-implantation. At 4 weeks post-implantation, channelled constructs stained homogenously for alizarin red, whereas the staining in solid constructs was heterogeneous, with the core region of the engineered tissue staining negatively, indicating that it was devoid of mineral. H&E staining of acellular (i.e. MSC-free) hydrogels 8 weeks post-implantation demonstrated no bone or cartilage tissue formation in either solid or channelled constructs, though small levels of cellular infiltration were apparent within the channels of channelled constructs (data not shown).

**Figure 4:**
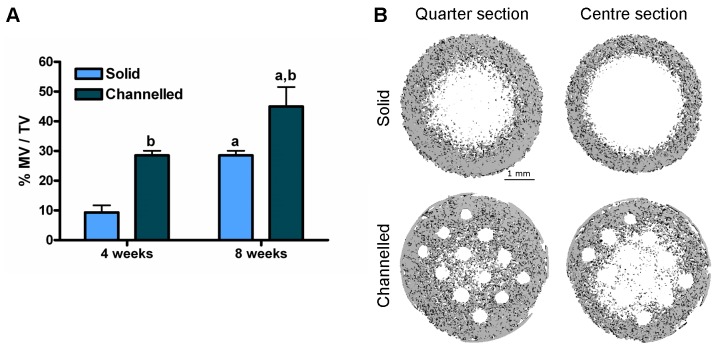
µCT analysis of constructs post-implantation. Constructs were analysed by µCT at 4 weeks and 8 weeks post-implantation to examine mineralisation. (A) % Mineral volume per total volume of construct (% MV/TV). 3 constructs per group were analysed. Significance *p*<0.05: a vs. 4 weeks post-implantation, b vs. solid constructs. (B) µCT images of constructs 8 weeks post-implantation showing spatial variance of mineralisation with depth through the construct. Quarter section corresponds to a region ∼0.75 mm into the depth of the construct. Centre section corresponds to a region ∼1.5 mm into the depth of the construct. Sections correspond to a thickness of 120 µm. Images are representative of 3 constructs analysed.

µCT analysis was performed on MSC-seeded hydrogels at 4 and 8 weeks post-implantation to assess the spatial distribution of mineral and it’s variance with depth through the construct, and also to quantify the volume of mineral in the construct. Mineral volume, as quantified by µCT, was significantly higher for channelled constructs as compared to solid constructs at both 4 and 8 weeks post-implantation (44.99±6.54 vs. 28.54±1.53% MV/TV, channelled constructs vs. solid constructs 8 weeks post-implantation, *p* = 0.014), see Fig. 4(A). At 8 weeks post-implantation, at a depth of ∼0.75 mm from the tissue surface, solid constructs had a core region devoid of mineral, whereas a homogenous deposition of mineral was observed in the channelled constructs, see Fig. 4(B). At a depth of ∼1.5 mm from the surface, the core region devoid of mineral in solid constructs had increased in area. While there was also evidence of a non-mineralised core region developing in channelled constructs, this area appeared smaller than the corresponding region in the solid constructs.

### Channels Act as Conduits for Vascularisation and Provide a Milieu for Endochondral Bone and Marrow Formation

**Figure 5:**
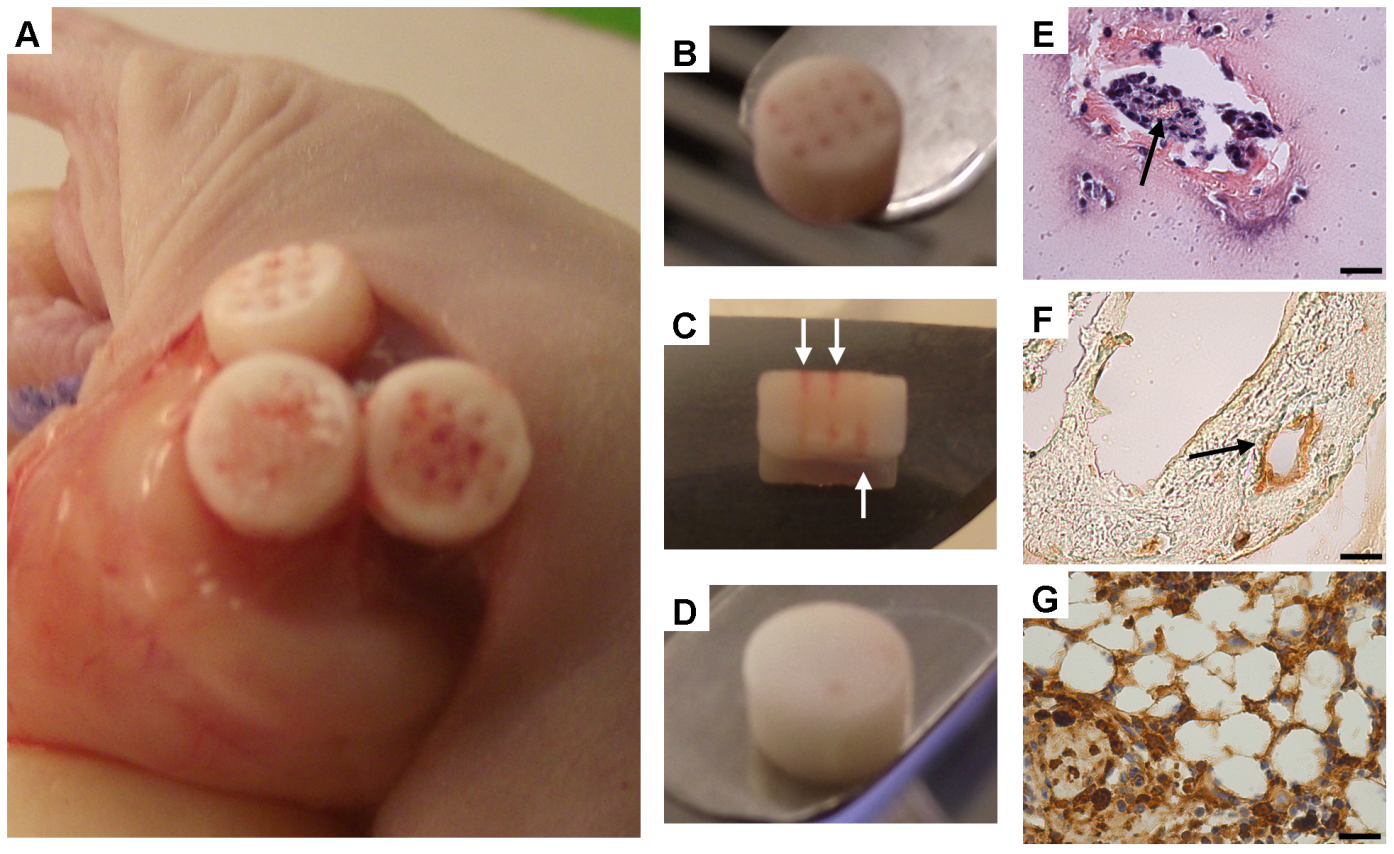
Vascularisation of channelled constructs post-implantation. (A) Retrieval of channelled constructs from the subcutaneous pocket 8 weeks post-implantation. (B) Image of a channelled construct 8 weeks post-implantation. (C) Image of a channelled construct sliced in half longitudinally to enable visualisation through the depth of the channels. White arrows indicate vascularised tissue within the channels. (D) Image of a solid construct 8 weeks post-implantation. (E) H&E staining of a channel 4 weeks post-implantation. (F) CD31 staining of a channel 4 weeks post-implantation. Black arrows indicate vessel-like structures. (G) TdT staining of a channel 4 weeks post-implantation. Brown and blue staining indicates cells positive and negative for TdT respectively. Scale bars are 25 µm.

Upon retrieval from the back of nude mice, macroscopically the channels appeared to the filled with a reddish, well vascularised tissue, see Fig. 5(A,B). This vascularised tissue spread through the depth of the channels, see Fig. 5(C). In contrast, macroscopically the solid constructs did not appear to be vascularised, see Fig. 5(D). Histological and immunohistochemical analysis was carried out on constructs to further investigate vascularisation and *de novo* tissue formation within the channels, and to determine the pathway through which this tissue formation occurs. At 4 weeks post-implantation channels were stained for H&E and for CD31 to examine the presence of blood vessels and endothelial cells. TdT staining was also carried out to assess the proportion of primitive marrow cells in the cellular marrow. Blood vessel structures were identified by H&E and CD31 staining, see Fig. 5(E,F). Vascularisation did not appear to progress significantly from the channels into the calcified cartilage within the hydrogel. TdT staining at 4 weeks post implantation indicated a scanty positive staining as expected for normal marrow, see Fig. 5(G).

**Figure 6:**
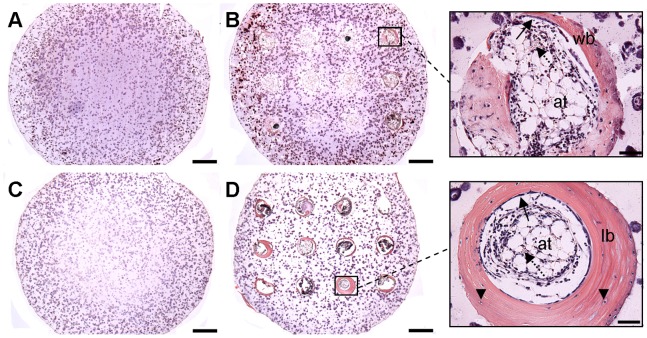
H&E staining of constructs post-implantation. Constructs were stained with H&E at 4 weeks and 8 weeks post-implantation to examine bone formation. (A) Solid construct 4 weeks post-implantation. (B) Channelled construct 4 weeks post-implantation. (C) Solid construct 8 weeks post-implantation. (D) Channelled construct 8 weeks post-implantation. Arrows show lining of osteoblasts laying down new bone, arrowheads show osteocytes embedded within bone matrix, dotted arrows show hematopoietic elements. ‘at’ – adipose tissue, ‘wb’ – woven bone, ‘lb’ – lamellar bone. Main image scale bars are 500 µm. Inset scale bars are 50 µm.

H&E staining of solid constructs did not demonstrate bone formation 4 weeks post-implantation, see Fig. 6(A). H&E staining of channelled constructs at 4 weeks post-implantation demonstrated woven bone formation within the channels, surrounding a marrow component consisting of a mixture of hematopoietic foci and marrow adipose tissue, see Fig. 6(B). At 8 weeks post-implantation no bone formation was evident in solid constructs, see Fig. 6(C), whereas within the channels of channelled constructs lamellar-like bone was evident, with the appearance of osteocyte-like cells embedded within the bone matrix, which again surrounded a marrow component, see Fig. 6(D).

**Figure 7:**
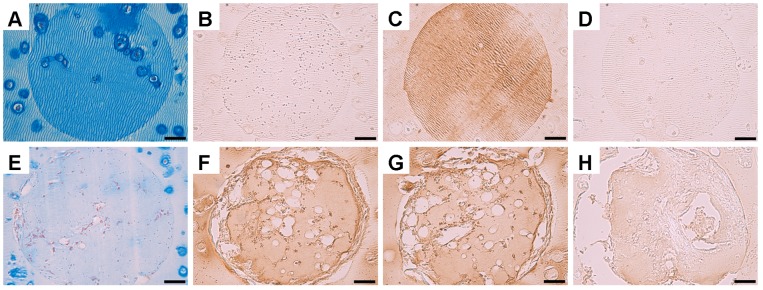
Histology and immunohistochemistry of the channels within channelled constructs pre-implantation and 4 weeks post-implantation. Channels were examined to determine the pathway through which bone formation occurs. (A,E) Alcian blue staining. (B,F) Collagen I staining. (C,G) Collagen II staining. (D,H) Collagen X staining. (A–D) Pre-implantation. (E–H) 4 weeks post-implantation. Scale bars are 50 µm.

Prior to subcutaneous implantation (i.e. during the *in vitro* priming phase), channels had partially filled with a cartilaginous matrix containing MSCs which stained intensely for alcian blue and collagen type II and negatively for collagens type I and X, see Fig. 7(A-D). At 4 weeks post-implantation channels stained very weakly for alcian blue, and very intensely for collagen type I, see Fig. 7(E,F). At this time point a decrease in the intensity of staining for collagen type II, as well as an increase in the intensity of staining for collagen type X, was also observed within channels as compared to pre-implantation levels, see Fig. 7(G,H). This suggests that the cartilage that had filled the channels *in vitro* supported endochondral bone formation *in vivo*.

## Discussion

New vessel formation is critical for bone tissue regeneration. During long bone growth, canals within the developing hypertrophic cartilage facilitate vascular invasion and the migration of osteogenic cells, thus playing a key role in endochondral ossification. The aim of this study was to mimic this function in tissue engineered hypertrophic cartilaginous constructs. We hypothesised that introducing an array of channels into MSC-seeded constructs would firstly facilitate the *in vitro* engineering of scaled-up hypertrophic cartilaginous tissues, and secondly, would accelerate the vascularisation and mineralisation of the engineered graft following implantation *in vivo*. During long-term *in vitro* culture, the presence of this array of channels lead to a reduction in total sGAG and collagen accumulation, whereas calcium deposition was enhanced in channelled constructs. In support of our hypothesis, the channels acted as conduits for blood vessel infiltration and their presence accelerated mineralisation of the engineered tissue *in vivo*. Finally, channels themselves provided a milieu for bone and marrow formation, with *de novo* bone being generated within these channels via the endochondral pathway.

Previous studies have investigated introducing nutrient channels into cartilaginous constructs in order to enhance matrix synthesis and the functional properties of tissue engineered cartilage [24]. For example, the introduction of a single nutrient channel into chondrocyte seeded agarose hydrogels results in superior mechanical properties and the formation of a more uniform fibrillar network whilst maintaining similar levels of sGAG and collagen content [22]. In the present study, scaffold architecture differentially regulated matrix synthesis, with solid constructs accumulating significantly more sGAG and collagen, and channelled constructs accumulating significantly more calcium. It may be that the channels provide a pathway for the diffusion of sGAGs and collagen out of the engineered tissue and into the media, and that calcium deposition is less influenced by such phenomena. Alternatively, or perhaps in conjunction, it may be that the altered rates of sGAG, collagen and calcium deposition in solid and channelled constructs is due to differences in the spatial gradients in regulatory cues that develop in these tissues. In solid hydrogels, the cellular consumption of oxygen at the periphery of the construct will result in the development of a low oxygen microenvironment within the core of the engineered tissue [37], [38], which has been shown to enhance sGAG and collagen synthesis [33], [39]. The introduction of channels into scaffolds may increase core oxygen levels [40], which has been shown to enhance hypertrophy and mineralisation of chondrogenically primed MSCs [33], [41].

Previous attempts to engineer scaled-up cartilaginous grafts for endochondral bone repair have reported the development of a core region devoid of cells and cartilaginous matrix [12]. This phenomenon was not observed in our study. However, if the endochondral approach is to be implemented to treat critically sized bone defects, further scaling-up of *in vitro* cartilaginous grafts will be required, and at these very large scales nutrient diffusion limitations are likely to develop within the construct. It is perhaps only at these dimensions, and where core degradation would otherwise occur, that the introduction of channels would benefit the *in vitro* development of an engineered cartilaginous tissue.

Following *in vivo* implantation, biochemical and histological analysis of MSC- seeded constructs revealed a reduction in sGAG content and an increase in calcium accumulation, indicating a loss of the chondrogenic phenotype and progression down the endochondral route. Interestingly, while solid constructs maintained their pre-implantation collagen levels during the course of the *in vivo* implantation period, channelled constructs continued to accumulate collagen, which may be due in part to the formation of new tissue within the channels themselves. Both histological data and the DNA assay suggested that channels became highly cellularised once implanted *in vivo*. While we cannot rule out a role played by donor cells, as there were MSCs present within channels pre-implantation, previous studies investigating bone tissue engineering *via* the endochondral pathway have highlighted an important role played by recruited host derived cells in driving endochondral ossification [10], [14]. It would appear that the channels in our tissue engineered hypertrophic cartilaginous constructs may be acting as conduits for cellular infiltration from the host, which are then playing a role in laying down new tissue within the construct.

In bone tissue engineering strategies, the prevention of core necrosis during *in vitro* development and following *in vivo* implantation is a significant challenge [5]. In the present study we utilised µCT to investigate the spatial variance of *in vivo* mineralisation with depth through the tissue and found that channelled architectures accelerated mineralisation, and furthermore, promoted the development of a more homogenous mineralised construct and limited the development of a core region devoid of viable cells and mineral. In addition to the inherent increase in nutrients and oxygen due to presence of the channels, they also facilitate blood vessel infiltration as evident by staining for CD31 positive endothelial cells, which in turn further increases oxygen levels and provides soluble factors and signals to promote osteogenesis [42].

Subcutaneous implantation of chondrogenically primed MSCs has been shown to produce endochondral bone containing hematopoietic marrow at 8 weeks post-implantation [8], [9]. In the present study, high magnification images of channels at 4 weeks post implantation demonstrated deposition of an immature woven bone, being laid down by osteoblasts, which at 8 weeks post-implantation had developed into lamellar-like bone with osteocyte-like cells evident within the bone matrix. Temporal analysis of chondrogenic extracellular matrix progression within channels indicated degradation of the sGAG/collagen type II rich-matrix following *in vivo* implantation, and a corresponding increase in the accumulation of collagen type I and collagen type X, suggesting that *de novo* bone found in the channels was being generated *via* the endochondral pathway. Moreover, at both *in vivo* time points there was evidence of hematopoietic foci and marrow adipose tissue, enclosed within the developing bone matrix. Given that it has previously been demonstrated that endochondral ossification is required for hematopoietic stem cell niche formation [43], this provides further validation that the mechanism through which bone is formed within the channels is indeed endochondral.

In the current study cartilaginous tissues were engineered using 4 month old, skeletally immature, porcine MSCs. Skeletally immature MSC seeded hydrogels have been shown to generate cartilaginous tissues with superior matrix deposition and mechanical properties as compared to skeletally mature MSC seeded hydrogels [44]. Further studies are required to confirm if the beneficial effect of channels, as shown in this study, would occur with the use of engineered tissues generated from a skeletally mature donor where the inherent regenerative capacity is not as high.

Bone tissue engineering *via* endochondral ossification has recently been demonstrated with collagen mesh scaffolds [12], on three-dimensional electrospun fibers [45], and with the bone void filler NuOss [46]. Utilising hydrogels as scaffolds for endochondral bone regeneration may be a particularly powerful tool in ‘scaling up’ tissue engineered grafts in order to treat defects of a clinically relevant size [38]. However, the chondrogenically primed MSC-seeded agarose hydrogels utilised in this study appeared to only partially support progression towards endochondral ossification, with the engineered tissue laid down in the main body of the hydrogel apparently locked within a hypertrophic, calcified cartilage state at 8 weeks post- implantation. Since the cartilaginous matrix within channels, which had filled up at least partially *in vitro* with scaffold-free tissue, had the ability to undergo full endochondral ossification *in vivo* it would appear that the agarose hydrogel acted as a barrier for vascularisation and the transition from mineralised cartilage to endochondral bone. This may be due to the agarose hydrogel undergoing minimal degradation during the 8 week *in vivo* period. A previous study investigating alginate hydrogels as scaffolds for bone tissue engineering demonstrated enhanced bone formation by accelerating the degradation properties of the hydrogel [47]. Future studies in our lab will examine the capacity of different naturally derived biodegradable hydrogels to produce endochondral bone *in vivo*, with the ultimate goal of scaling up anatomically accurate cartilaginous grafts as a paradigm for whole bone tissue engineering via endochondral ossification. However, all scaffolds or hydrogels will likely impede vascularisation to some degree, and adopting the channel strategy developed in this study is expected to accelerate the process of endochondral ossification regardless of the hydrogel used to tissue engineer hypertrophic cartilage grafts for bone regeneration.

### Conclusion

Inspired by the cartilage canal network observed during endochondral skeletogenesis, this study demonstrated that tissue engineering channelled hypertrophic cartilaginous constructs accelerates mineralisation and vascularisation of the graft *in vivo* and shows promise for use in future endochondral bone regeneration strategies. The study reinforces the importance of optimising the architecture of engineered constructs targeting bone tissue regeneration, even if this is achieved via an endochondral pathway as opposed to the traditional intramembranous route.

## References

[1] Langer R (2000) Tissue Engineering. Molecular Therapy 1: 12–15. 10933907 10.1006/mthe.1999.0003

[2] Koh CJ, Atala A (2004) Tissue Engineering, Stem Cells, and Cloning: Opportunities for Regenerative Medicine. Journal of the American Society of Nephrology 15: 1113–1125. 15100351 10.1097/01.asn.0000119683.59068.f0

[3] Meijer GJ, De Bruijn JD, Koole R, Van Blitterswijk CA (2007) Cell-based bone tissue engineering. PLoS Medicine 4: 0260–0264. 10.1371/journal.pmed.0040009 PMC1800310 17311467

[4] Santos MI, Reis RL (2010) Vascularization in bone tissue engineering: Physiology, current strategies, major hurdles and future challenges. Macromolecular Bioscience 10: 12–27. 19688722 10.1002/mabi.200900107

[5] Lyons FG, Al-Munajjed AA, Kieran SM, Toner ME, Murphy CM, et al (2010) The healing of bony defects by cell-free collagen-based scaffolds compared to stem cell-seeded tissue engineered constructs. Biomaterials 31: 9232–9243. 20863559 10.1016/j.biomaterials.2010.08.056

[6] Jukes JM, Both SK, Leusink A, Sterk LMT, Van Blitterswijk CA, et al (2008) Endochondral bone tissue engineering using embryonic stem cells. Proceedings of the National Academy of Sciences of the United States of America 105: 6840–6845. 18467492 10.1073/pnas.0711662105 PMC2374550

[7] Farrell E, Van Der Jagt OP, Koevoet W, Kops N, Van Manen CJ, et al (2009) Chondrogenic priming of human bone marrow stromal cells: A better route to bone repair? Tissue Eng Part C Methods 15: 285–295. 19505182 10.1089/ten.tec.2008.0297

[8] Scotti C, Tonnarelli B, Papadimitropoulos A, Scherberich A, Schaeren S, et al (2010) Recapitulation of endochondral bone formation using human adult mesenchymal stem cells as a paradigm for developmental engineering. Proceedings of the National Academy of Sciences of the United States of America 107: 7251–7256. 20406908 10.1073/pnas.1000302107 PMC2867676

[9] Janicki P, Kasten P, Kleinschmidt K, Luginbuehl R, Richter W (2010) Chondrogenic pre-induction of human mesenchymal stem cells on β-TCP: Enhanced bone quality by endochondral heterotopic bone formation. Acta Biomaterialia 6: 3292–3301. 20123138 10.1016/j.actbio.2010.01.037

[10] Tortelli F, Tasso R, Loiacono F, Cancedda R (2010) The development of tissue-engineered bone of different origin through endochondral and intramembranous ossification following the implantation of mesenchymal stem cells and osteoblasts in a murine model. Biomaterials 31: 242–249. 19796807 10.1016/j.biomaterials.2009.09.038

[11] Lau TT, Lee LQP, Vo BN, Su K, Wang DA (2012) Inducing ossification in an engineered 3D scaffold-free living cartilage template. Biomaterials 33: 8406–8417. 22925815 10.1016/j.biomaterials.2012.08.025

[12] Scotti C, Piccinini E, Takizawa H, Todorov A, Bourgine P, et al (2013) Engineering of a functional bone organ through endochondral ossification. Proceedings of the National Academy of Sciences of the United States of America 110: 3997–4002. 23401508 10.1073/pnas.1220108110 PMC3593845

[13] Sheehy EJ, Vinardell T, Buckley CT, Kelly DJ (2013) Engineering osteochondral constructs through spatial regulation of endochondral ossification. Acta Biomaterialia 9: 5484–5492. 23159563 10.1016/j.actbio.2012.11.008

[14] Farrell E, Both SK, Odörfer KI, Koevoet W, Kops N, et al.. (2011) In-vivo generation of bone via endochondral ossification by in-vitro chondrogenic priming of adult human and rat mesenchymal stem cells. BMC Musculoskeletal Disorders 12. 10.1186/1471-2474-12-31 PMC3045394 21281488

[15] Pelttari K, Winter A, Steck E, Goetzke K, Hennig T, et al (2006) Premature induction of hypertrophy during in vitro chondrogenesis of human mesenchymal stem cells correlates with calcification and vascular invasion after ectopic transplantation in SCID mice. Arthritis and Rheumatism 54: 3254–3266. 17009260 10.1002/art.22136

[16] Gerber HP, Vu TH, Ryan AM, Kowalski J, Werb Z, et al (1999) VEGF couples hypertrophic cartilage remodeling, ossification and angiogenesis during endochondral bone formation. Nat Med 5: 623–628. 10371499 10.1038/9467

[17] Lee CH, Marion NW, Hollister S, Mao JJ (2009) Tissue formation and vascularization in anatomically shaped human joint condyle ectopically in vivo. Tissue Engineering - Part A 15: 3923–3930. 19563263 10.1089/ten.tea.2008.0653 PMC2792071

[18] Martin I, Wendt D, Heberer M (2004) The role of bioreactors in tissue engineering. Trends Biotechnol 22: 80–86. 14757042 10.1016/j.tibtech.2003.12.001

[19] Mauck RL, Soltz MA, Wang CCB, Wong DD, Chao PHG, et al (2000) Functional tissue engineering of articular cartilage through dynamic loading of chondrocyte-seeded agarose gels. Journal of Biomechanical Engineering 122: 252–260. 10923293 10.1115/1.429656

[20] Mauck RL, Nicoll SB, Seyhan SL, Ateshian GA, Hung CT (2003) Synergistic action of growth factors and dynamic loading for articular cartilage tissue engineering. Tissue Eng 9: 597–611. 13678439 10.1089/107632703768247304

[21] Thorpe SD, Buckley CT, Vinardell T, O’Brien FJ, Campbell VA, et al (2010) The response of bone marrow-derived mesenchymal stem cells to dynamic compression following tgf-β3 induced chondrogenic differentiation. Annals of Biomedical Engineering 38: 2896–2909. 20458627 10.1007/s10439-010-0059-6

[22] Bian L, Angione SL, Ng KW, Lima EG, Williams DY, et al (2009) Influence of decreasing nutrient path length on the development of engineered cartilage. Osteoarthritis Cartilage 17: 677–685. 19022685 10.1016/j.joca.2008.10.003 PMC3387279

[23] Zhang Y, Yang F, Liu K, Shen H, Zhu Y, et al (2012) The impact of PLGA scaffold orientation on invitro cartilage regeneration. Biomaterials 33: 2926–2935. 22257722 10.1016/j.biomaterials.2012.01.006

[24] Buckley CT, Thorpe SD, Kelly DJ (2009) Engineering of large cartilaginous tissues through the use of microchanneled hydrogels and rotational culture. Tissue Engineering - Part A 15: 3213–3220. 19374490 10.1089/ten.TEA.2008.0531

[25] Sheehy EJ, Buckley CT, Kelly DJ (2011) Chondrocytes and bone marrow-derived mesenchymal stem cells undergoing chondrogenesis in agarose hydrogels of solid and channelled architectures respond differentially to dynamic culture conditions. Journal of Tissue Engineering and Regenerative Medicine 5: 747–758. 21953872 10.1002/term.385

[26] Mesallati T, Buckley CT, Nagel T, Kelly DJ (2012) Scaffold architecture determines chondrocyte response to externally applied dynamic compression. Biomechanics and Modeling in Mechanobiology: 1–11. 10.1007/s10237-012-0451-2 23160843

[27] Ganey TM, Ogden JA, Sasse J, Neame PJ, Hilbelink DR (1995) Basement membrane composition of cartilage canals during development and ossification of the epiphysis. Anatomical Record 241: 425–437. 7755183 10.1002/ar.1092410318

[28] Ytrehus B, Carlson CS, Lundeheim N, Mathisen L, Reinholt FP, et al (2004) Vascularisation and osteochondrosis of the epiphyseal growth cartilage of the distal femur in pigs - Development with age, growth rate, weight and joint shape. Bone 34: 454–465. 15003793 10.1016/j.bone.2003.07.011

[29] Blumer MJF, Longato S, Fritsch H (2008) Structure, formation and role of cartilage canals in the developing bone. Annals of Anatomy 190: 305–315. 18602255 10.1016/j.aanat.2008.02.004

[30] Blumer MJF, Schwarzer C, Pérez MT, Konakci KZ, Fritsch H (2006) Identification and location of bone-forming cells within cartilage canals on their course into the secondary ossification centre. Journal of Anatomy 208: 695–707. 16761972 10.1111/j.1469-7580.2006.00578.x PMC2100231

[31] Lenas P, Moos M, Luyten FP (2009) Developmental engineering: A new paradigm for the design and manufacturing of cell-based products. Part I: From three-dimensional cell growth to biomimetics of in Vivo development. Tissue Engineering - Part B: Reviews 15: 381–394. 19505199 10.1089/ten.TEB.2008.0575

[32] Lennon DP, Caplan AI (2006) Isolation of human marrow-derived mesenchymal stem cells. Exp Hematol 34: 1604–1605. 17046583 10.1016/j.exphem.2006.07.014

[33] Sheehy EJ, Buckley CT, Kelly DJ (2012) Oxygen tension regulates the osteogenic, chondrogenic and endochondral phenotype of bone marrow derived mesenchymal stem cells. Biochemical and Biophysical Research Communications 417: 305–310. 22155244 10.1016/j.bbrc.2011.11.105

[34] Vinardell T, Sheehy EJ, Buckley CT, Kelly DJ (2012) A comparison of the functionality and in vivo phenotypic stability of cartilaginous tissues engineered from different stem cells sources. Tissue Engineering Part A 18: 1161–1170. 22429262 10.1089/ten.tea.2011.0544 PMC3360504

[35] Kafienah W, Sims TJ (2004) Biochemical methods for the analysis of tissue-engineered cartilage. Methods Mol Biol 238: 217–230. 14970450 10.1385/1-59259-428-x:217

[36] Ignat’eva NY, Danilov NA, Averkiev SV, Obrezkova MV, Lunin VV, et al (2007) Determination of hydroxyproline in tissues and the evaluation of the collagen content of the tissues. J Anal Chem 62: 51–57.

[37] Thorpe SD, Nagel T, Carroll SF, Kelly DJ (2013) Modulating Gradients in Regulatory Signals within Mesenchymal Stem Cell Seeded Hydrogels: A Novel Strategy to Engineer Zonal Articular Cartilage. PLoS ONE 8. 10.1371/journal.pone.0060764 PMC3628868 23613745

[38] Buckley CT, Meyer EG, Kelly DJ (2012) The influence of construct scale on the composition and functional properties of cartilaginous tissues engineered using bone marrow-derived mesenchymal stem cells. Tissue Engineering - Part A 18: 382–396. 21919793 10.1089/ten.TEA.2011.0145

[39] Meyer EG, Buckley CT, Thorpe SD, Kelly DJ (2010) Low oxygen tension is a more potent promoter of chondrogenic differentiation than dynamic compression. Journal of Biomechanics 43: 2516–2523. 20557888 10.1016/j.jbiomech.2010.05.020

[40] Buckley CT, O’Kelly KU (2010) Fabrication and characterization of a porous multidomain hydroxyapatite scaffold for bone tissue engineering investigations. Journal of Biomedical Materials Research - Part B Applied Biomaterials 93: 459–467. 10.1002/jbm.b.31603 20166121

[41] Gawlitta D, Van Rijen MHP, Schrijver EJM, Alblas J, Dhert WJA (2012) Hypoxia impedes hypertrophic chondrogenesis of human multipotent stromal cells. Tissue Engineering - Part A 18: 1957–1966. 22563686 10.1089/ten.TEA.2011.0657

[42] Brandi ML, Collin-Osdoby P (2006) Vascular biology and the skeleton. Journal of Bone and Mineral Research 21: 183–192. 16418774 10.1359/JBMR.050917

[43] Chan CKF, Chen CC, Luppen CA, Kim JB, DeBoer AT, et al (2009) Endochondral ossification is required for haematopoietic stem-cell niche formation. Nature 457: 490–494. 19078959 10.1038/nature07547 PMC2648141

[44] Erickson IE, Van Veen SC, Sengupta S, Kestle SR, Mauck RL (2011) Cartilage matrix formation by bovine mesenchymal stem cells in three-dimensional culture is age-dependent. Clinical Orthopaedics and Related Research 469: 2744–2753. 21424832 10.1007/s11999-011-1869-z PMC3171558

[45] Yang W, Yang F, Wang Y, Both SK, Jansen JA (2013) In vivo bone generation via the endochondral pathway on three-dimensional electrospun fibers. Acta Biomaterialia 9: 4505–4512. 23059416 10.1016/j.actbio.2012.10.003

[46] Weiss HE, Roberts SJ, Schrooten J, Luyten FP (2012) A semi-autonomous model of endochondral ossification for developmental tissue engineering. Tissue Engineering - Part A 18: 1334–1343. 22394057 10.1089/ten.TEA.2011.0602

[47] Simmons CA, Alsberg E, Hsiong S, Kim WJ, Mooney DJ (2004) Dual growth factor delivery and controlled scaffold degradation enhance in vivo bone formation by transplanted bone marrow stromal cells. Bone 35: 562–569. 15268909 10.1016/j.bone.2004.02.027

